# Modeling Rheumatoid Arthritis In Vitro: From Experimental Feasibility to Physiological Proximity

**DOI:** 10.3390/ijms21217916

**Published:** 2020-10-25

**Authors:** Alexandra Damerau, Timo Gaber

**Affiliations:** 1 Charité—Universitätsmedizin Berlin, corporate member of Freie Universität Berlin, Humboldt-Universität zu Berlin, and Berlin Institute of Health, Department of Rheumatology and Clinical Immunology, 10117 Berlin, Germany; alexandra.damerau@charite.de; 2 German Rheumatism Research Centre (DRFZ) Berlin, a Leibniz Institute, 10117 Berlin, Germany

**Keywords:** in vitro models, rheumatoid arthritis, cytokines, mesenchymal stromal cells, co-culture, tissue engineering, 3D cell culture, explants, joint-on-a-chip

## Abstract

Rheumatoid arthritis (RA) is a chronic, inflammatory, and systemic autoimmune disease that affects the connective tissue and primarily the joints. If not treated, RA ultimately leads to progressive cartilage and bone degeneration. The etiology of the pathogenesis of RA is unknown, demonstrating heterogeneity in its clinical presentation, and is associated with autoantibodies directed against modified self-epitopes. Although many models already exist for RA for preclinical research, many current model systems of arthritis have limited predictive value because they are either based on animals of phylogenetically distant origin or suffer from overly simplified in vitro culture conditions. These limitations pose considerable challenges for preclinical research and therefore clinical translation. Thus, a sophisticated experimental human-based in vitro approach mimicking RA is essential to (i) investigate key mechanisms in the pathogenesis of human RA, (ii) identify targets for new therapeutic approaches, (iii) test these approaches, (iv) facilitate the clinical transferability of results, and (v) reduce the use of laboratory animals. Here, we summarize the most commonly used in vitro models of RA and discuss their experimental feasibility and physiological proximity to the pathophysiology of human RA to highlight new human-based avenues in RA research to increase our knowledge on human pathophysiology and develop effective targeted therapies.

## 1. Introduction

**Figure 1:**
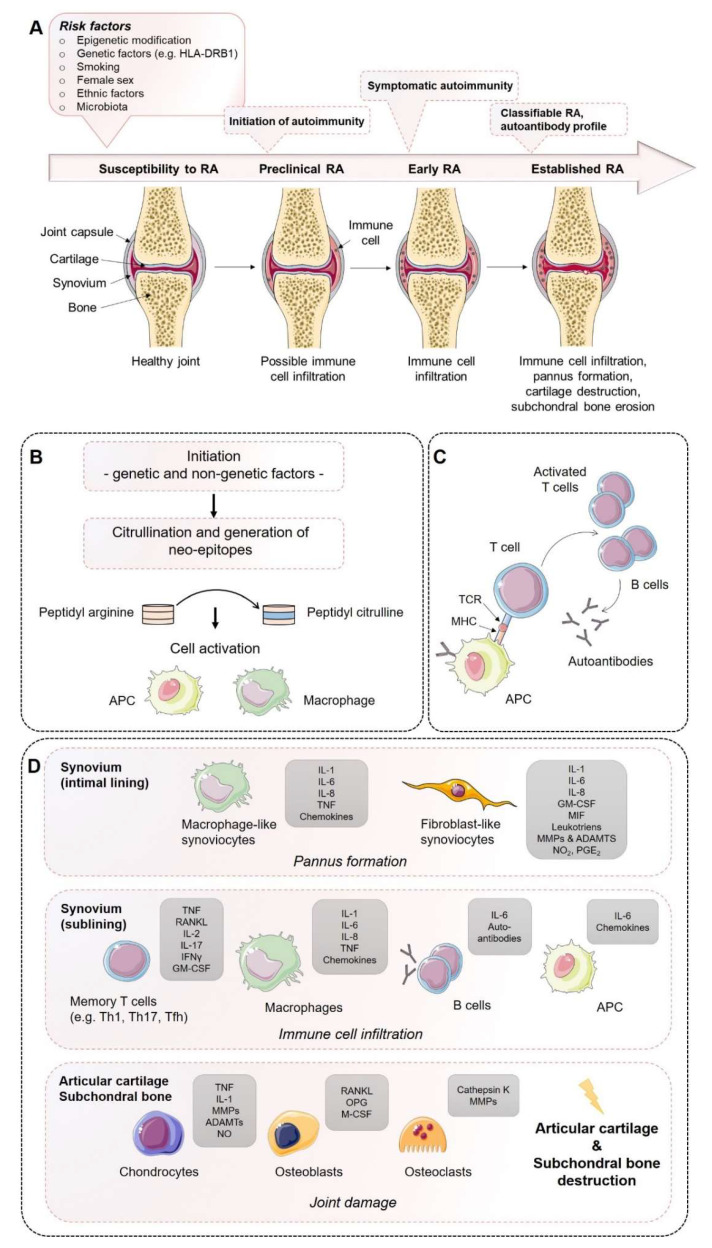
Establishment of rheumatoid arthritis (RA): Mechanisms of disease initiation, development, and progression. (**A**) Multiple risk factors, including both genetic and non-genetic influences, are required to induce the development of RA in susceptible individuals. Years before first clinical symptoms of RA occur, autoimmunity against modified self-proteins is initiated, which results in the onset of a subclinical inflamed synovium (symptomatic autoimmunity) propagated by immune cell infiltration and pannus formation. Once established, RA can be classified according to the clinical symptoms. (**B**) Onset of autoimmunity is supposed to occur in the mucosa (e.g., mouth, lung, and gut) by the creation of neo-epitopes as a result of post-translational modifications, e.g., by citrullination. These neo-epitopes can be recognized by antigen-presenting cells (APCs) of the adaptive immune system and (**C**) are presented to adaptive immune cells in lymphoid tissues, activate an immune response, and induce autoantibody formation (e.g., ACPA and RF). (**D**) Activated immune cells and immune complexes can activate synovial cells, such as fibroblast-like synoviocytes (FLS) and macrophage-like synoviocytes of the intimal lining and APCs in the sublining area, to produce a range of inflammatory factors and expand and form the cartilage- and bone-invasive pannus. Autoimmune activation and immune cell infiltration (T cells, B cells, macrophages) of the sublining area further contribute to the excessive production of inflammatory factors, autoantibodies, and synovial vascular leakage, ultimately leading to articular cartilage and subchondral bone destruction as a result of matrix-degrading enzymes and a de-balanced bone homeostasis characterized by an imbalanced RANKL/RANK/OPG system and activated osteoclasts. ADAMTS, a disintegrin and metalloproteinase with thrombospondin motifs; APCAs, anti-citrullinated protein antibodies; RF, rheumatoid factor; GM-CSF, granulocyte–macrophage colony-stimulating factor; M-CSF, macrophage colony-stimulating factor; MHC, major histocompatibility complex; MMP, matrix metalloproteinase; NO, nitric oxide; OPG, osteoprotegerin; RANKL, receptor activator of nuclear factor-κB ligand; RANK, receptor activator of nuclear factor-κB; TCR, T cell receptor; TNF, tumor necrosis factor. Figure contains graphics from Servier Medical Art, licensed under a Creative Common Attribution 3.0 Generic License. http://smart.servier.com/.

Rheumatoid arthritis (RA) is a progressive systemic, chronic, and inflammatory autoimmune disease with an average prevalence of 0.5–1.0% in the population worldwide, demonstrating ethnic and geographic differences [1]. Its pathogenesis is characterized by immune cell infiltration into the synovial membrane and the joint cavity and the formation of hyperplastic and invasive synovium, resulting in progressive cartilage destruction and subchondral bone erosion in late stages of disease if not treated (Figure 1). Along with the joints, RA can affect many of the body’s organs, including the heart, eyes, skin, intestine, kidney, lung, and brain, as well as the skeleton [2,3]. A disease most likely RA was first recognized more than 20 centuries ago as a disease that painfully affects the body’s joints [4]. It is the most common inflammatory joint disease affecting both individuals and society. The affected patients suffer a considerable loss of quality of life and a decline in productivity, and the effort and costs of health care increase, ultimately resulting in a major economic and social burden [5]. Symptoms of RA most commonly include pain, swelling, and morning stiffness in the affected joints. It is a multifactorial disorder and recent studies have identified multiple genetic and environmental factors associated with an increased risk of RA, e.g., female sex, smoking, and major histocompatibility complex (MHC) regions encoding human leukocyte antigen (HLA) proteins (amino acids at positions 70 and 71) [2,6]. Years before first clinical symptoms of RA occur, autoimmunity against modified self-proteins is initiated, which results in the onset of the disease [1].

As the course of RA within the individual patients may differ with regard to pathogenesis, clinical symptoms, and diseases subtypes, personalized precision medicine must be the ultimate goal to achieve disease remission. To date, we are far from curing RA in part due to the need for (i) objective patient-related biomarkers to identify disease subtypes and treatment response and (ii) the management of patients who are refractory or resistant to available treatments. Having both will enable us to understand the disease and their pathogenic processes to optimize and introduce personalized precision health care.

## 2. The Course of RA Pathogenesis

The course of RA pathogenesis involves several stages. Before clinical symptoms are established, a certain level of RA susceptibility (e.g., genetic factors) coupled with the accumulation of risk factors proceed through the pre-clinical stage of the disease, leading to synovial inflammation, which, if not resolved, ultimately leads to the development of RA. During the early development of RA, post-translational modifications of a wide range of cellular (e.g., collagen) and nuclear proteins (e.g., histones) occur, including the conversion of the amino acid arginine to citrulline, a process called citrullination. Citrullination may be a result of smoking on mucosa, induced by microbiota (e.g., *Porphyromonas gingivalis*) or by an overarching neutrophil reaction. Altered modified self-proteins engage professional antigen presenting cells (APCs), such as macrophages, as foreign and induce a normal immune response via the help of T cells, thereby stimulating B cells to produce a wide range of (auto)antibodies recognizing self-proteins, such as rheumatoid factor (RF) and anti-citrullinated protein antibodies (ACPAs). The presence of autoantibodies often occurs before the onset of clinical synovitis, leading to the assumption that a second not-fully-understood mechanism seems to be necessary for the transition of autoimmunity to local synovial inflammation [1,2,6].

However, during the progression of RA, increase in vascular permeability, a disrupted extracellular matrix, and synovial immune cell infiltration transform the paucicellular synovium into chronically inflamed tissue. This process includes the expansion of the intimal lining and activation of macrophage- and stromal-fibroblast-like synoviocytes (FLSs), which then produce a variety of pro-inflammatory humoral mediators, such as cytokines and chemokines, including interleukin (IL)-1β, IL-6, IL-8, tumor necrosis factor (TNF), granulocyte–macrophage colony-stimulating factor (GM-CSF), macrophage migration inhibitory factor (MIF), and matrix-degrading enzymes, e.g., matrix metallopeptidases (MMPs) and a disintegrin and metalloproteinase with thrombospondin motifs (ADAMTs), prostaglandins, leukotrienes, and reactive nitric oxide. The aggressive and invasive phenotype of expanding FLSs, forming the hyperplastic pannus tissue, contributes to cartilage damage but may also be responsible for propagation and systemic spreading of inflammation by migrating from joint to joint and other organs [7,8,9].

The inflammation-induced expansion of FLSs and the infiltration of inflammatory cells into the usually paucicellular synovium lead to an enhanced metabolic need and, therefore, to an undersupply of both nutrients and oxygen to the synovial tissue. Due to the resulting local hypoxia, new vessels are formed that further facilitate the inflammatory process by increasing the amount of adaptive immune cells, and especially CD4+ memory T (Th) cells infiltrating the synovial sublining. Lymphocyte infiltrates accumulate and form aggregates ranging from small and loosely arranged lymphocyte clusters to large and organized ectopic lymphoid structures, which, in some cases, develop germinal centers that facilitate local T cell–B cell interactions. In these ectopic germinal structures, specific pathologic follicular helper T cells (Tfh) promote B-cell responses and (auto)antibody production within pathologically-inflamed non-lymphoid tissues. Apart from pathogenic Tfh cells, Th1 and Th17 cells have been identified in the pathogenesis of RA. Although the evidence of the pathogenic function of Th1 cells in RA is controversial due to the lack of therapeutic efficiency targeting of interferon (IFN)-γ [10,11], it should be noted that biologic targeting of TNF-α, which is a Th1 cytokine, are successful treatments in RA [11]. An effect that can be explained by the suppressive nature of Th1 on Th17 is that the responses contribute to tissue damage through production of TNF and GM-CSF [12].

IL-17-producing CD4+ T cells have been identified in synovial tissues from patients with RA, including their inducing cytokines IL-6 IL-1β, IL-21, transforming growth factor (TGF)-β, and IL-23 [13,14,15,16,17], and have been demonstrated to be increased/maintained in the peripheral blood of RA patients [18,19,20,21], whereas IL-17 was shown to induce bone resorption and contribute to neutrophil recruitment, and particularly into the synovial fluid, a hallmark of RA [22,23]. Besides effector T helper cells, antigen-presenting follicular dendritic cells, macrophages, and mast cells are present in the synovial sublining and contribute to the chronic inflammation by a large number of inflammatory mediators, such as cytokines, chemokines, and reactive oxygen and nitrogen species, as well as matrix-degrading enzymes. In contrast, neutrophils are lacking in the inflamed synovial lining and sublining but are abundantly present in the synovial fluid. Recent studies proposed that distinct subtypes of synovial histology displaying inflammatory versus non-inflammatory patterns are associated with different clinical phenotypes and a concurring response to novel targeted therapeutic interventions [24,25]. Technical progression and the development and combination of state-of-the-art methods from single cell genomics to mass cytometry have provided new insights into the complex interplay of cells and soluble immune mediators, particularly cytokines and chemokines [2]. Thus, specific pathogenic infiltrating immune cell subsets—such as IL-1β positive pro-inflammatory monocytes, autoimmune-associated B cells, and peripheral helper T (Tph) cells sharing similarities with Tfh cells, distinct subsets of CD8+ T cells, as well as mast cells—contribute to the inflammatory pattern of the RA synovial lining/sublining [26,27,28,29,30,31].

Invading immune cells and FLSs of the synovial lining produce large amounts of pro-inflammatory cytokines and express high levels of MMPs, while the expression of endogenous MMP inhibitors remains insufficiently low. Finally, the invasive and destructive FLS-front of synovial tissue, called the pannus, attaches to the articular surface and contributes to local matrix destruction and cartilage degradation. The chondrocytes of the damaged articular cartilage contribute to the vicious cycle of cartilage degeneration by inducing inflammatory cytokines, such as IL-1β and TNF-α, as well as MMPs and nitric oxide (NO). Additionally, FLSs negatively affect the subchondral bone by activation and maturation of bone-resorbing osteoclasts. Osteoclasts are highly responsive to autoantibodies; pro-inflammatory cytokines, in particular TNF-α, IL-1β, and IL-6; and more importantly, receptor activator of nuclear factor kappa B ligand (RANKL), which is the key regulator of osteoclastogenesis. RANKL binds to its receptor, the receptor activator of nuclear factor-κB (RANK), and activates osteoclasts, leading to an enhancement of bone resorption. Conversely, osteoblasts that play a key role in the regulation of anabolic bone metabolism produce bone matrix constituents, induce bone matrix mineralization, and modulate osteoclasts through the production of osteoprotegerin (OPG) [32]. Although osteoblasts producing OPG, which is a decoy receptor for RANKL, results in protection from bone destruction by osteoclasts, they also generate RANKL and M-CSF, both of which contribute to osteoclastogenesis. Imbalanced bone remodeling both in the subchondral and periarticular bone of joints leads to bone erosions and periarticular osteopenia; generalized bone loss is a general feature of established RA.

## 3. Lessons from Animal Models of Arthritis: None are Truly RA

Animal models represent an integral part of the preclinical drug discovery process and are used to study pathophysiological mechanisms of RA. Despite their extreme usefulness for testing new approaches of intervention in many cases, concerns about low clinical development success rates for investigational drugs have been raised [33], “Dozens of preclinical arthritis models have been developed…none of these, however, is truly RA, and none consistently predicts the effect of a therapeutic agent in patients” [33].

Importantly, animals do not naturally develop autoimmune disorders, such as RA, which is an inherent limitation of these arthritis models (Table 1). Instead, animal models can be used to study certain specific pathophysiological aspects of human disease, such as destructive pathways involved in the erosion of articular cartilage and bone. To this end, arthritis can be chemically induced in these animals by soluble agents (e.g., type II collagen-induced arthritis model) or develop spontaneously after genetic manipulation (e.g., human TNF transgene model) (Table 1) [34,35,36]. Although most of these models display features of human RA, such as inflammatory cell infiltrate, synovial hyperplasia, pannus formation, cartilage destruction, and bone erosions, they also demonstrate specific limitations, such as the development of self-limiting arthritis, development of arthritis only in susceptible strains of rodents, and a pathophysiology that does not recapitulate the endogenous breach of tolerance and excludes systemic components of disease [34,35,36]. The mutations used in genetically engineered arthritis models have not been identified in human RA [36]. When comparing transcriptional programs of mice and humans overlapping but notably different gene expression patterns have been observed [37]. Therefore, therapeutic approaches, such as the application of biologics highly specific for human target proteins, cannot be proven using non-humanized rodent models [38]. Finally, mice and humans differ in their locomotion, life span, evolutionary pressures, ecological niches, circadian rhythms, weight bearing, and blood leukocyte population ratios. Thus, none of the animal models is capable of fully replicating human pathogenesis of RA, which provides an explanation for the observed challenges in clinical translation [33].

Modern management guidelines recommend early and rigorous treatment to achieve low disease activity or remission targets as rapidly as possible. Thus, RA is currently treated with a wide variety of therapeutic drugs ranging from steroidal/nonsteroidal anti-inflammatory drugs (NSAID), glucocorticoids (GCs), and disease-modifying anti-rheumatic drugs (DMARDs) of synthetic origin, such as conventional synthetic DMARDs (e.g., methotrexate), biological, and biosimilar DMARDs (e.g., TNF inhibitors or IL-6 inhibitors), as well as targeted synthetic DMARDs (the Janus kinase (JAK) inhibitors) targeting specific immune cells, cytokines, or pro-inflammatory pathways [2,26,39]. Today’s therapeutic approaches using state-of-the-art biologicals or JAK inhibitors have been proven to be highly successful and effective in most patients with RA, including those with severe disease progression. Despite major progress in the treatment of RA, a strong unmet medical need remains, as not all patients reach sustained clinical remission (less than half of patients with RA) and about 25% still suffer from moderate or even high disease activity [2,40]. Defining patients with RA (i) refractory to available treatments among patients with RA who are undertreated or non-adherent to treatment, (ii) identifying objective biomarkers for disease states (e.g., early versus established RA) and/or (iii) ‘refractory’ states and finally (iv) for states treatment response is still the greatest unmet need in RA [40]. The lack of therapeutic efficacy in the true refractory patients may be due to the nature of the “one-fits-it-all” approach of standardized therapeutic regimes. Thus, clinical management of patients often neglects their heterogeneity with regard to the endogenous circadian rhythms, disease states, subtypes and duration, as well as autoantibody, cytokine, and infiltrating immune cell pattern. Identifying objective biomarkers to delineate disease subtypes and treatment response will be necessary to provide a ‘precise’ customized treatment strategy for each individual patient enhancing our repertoire in the battle against this potentially devastating disease.

**Table 1:** Selected rodent models for rheumatoid arthritis (as reviewed in Reference [34,35,36]).

Animal Models for Rheumatoid Arthritis	Species	Induction/Genetic Alteration	Limitations	References
***Induced Arthritis Models***				
Collagen-induced arthritis (CIA)	Mouse, rat	Inoculation with type II heterologous or homologous collagen in complete Freund’s adjuvant in strains expressing major histocompatibility complex (MHC) Class II I-Aq haplotypes	▪ General variable incidence, severity, and inter-group inconsistency ▪ Only inducible in susceptible strains of rodents ▪ Low incidence, as well as variability, of arthritis severity in c57bl/6 mice ▪ Acute and self-limiting polyarthritis in contrast to human RA ▪ Greater incidence in males in contrast to human RA	[36,41,42,43]
Collagen-antibody-induced arthritis (CAIA)	Mouse	Anti-collagen antibodies have been demonstrated to induce arthritis	▪ Pathogenesis is not mediated via T and B cell response in contrast to human RA ▪ Pathogenesis is inducible irrespective of the presence of MHC class II haplotype in contrast to human RA	[44,45]
Adjuvant-induced arthritis (AA)	Mouse, rat	Mixture of mineral oils, heat-killed mycobacteria, and emulsifying agent, which was termed complete Freund’s adjuvant (CFA); when omitting mycobacteria, also known as incomplete Freund’s adjuvant (IFA); see also pristane-induced arthritis (PIA)	▪ Acute and self-limiting polyarthritis in contrast to human RA ▪ Not antigenic but displays an autoimmune pathophysiology	[34,35,46,47]
Zymosan-induced arthritis	Mouse, rat	Intra-articular injection of zymosan, a polysaccharide from the cell wall of *Saccharomyces cerevisiae*, into the knee joints of mice causes proliferative arthritis, including immune cell infiltration, synovial hypertrophy, and pannus formation	▪ Technical skill required for an intra-articular injection in mice ▪ Monoarthritis in contrast to human RA	[48,49]
Streptococcal cell-wall-induced arthritis (SCWIA)	Mouse, rat	*Streptococcus pyogenes* synthesize a peptidoglycan-polysaccharide (PG-PS) polymer	▪ Pathogenesis is inducible in selected susceptible strains of rodents ▪ Germ-free conditions are necessary to reach susceptibility in rats ▪ Multiple injections are needed; otherwise, acute and self-limiting arthritis develops, in contrast to human RA ▪ Tumor necrosis factor (TNF)-α is less important in SCW-induced arthritis but not in human RA ▪ Rheumatoid factor is missing in polyarticular arthritis in rats	[35,50,51]
Cartilage oligomeric matrix protein (COMP)-induced arthritis	Mouse, rat	Immunization with IFA combined with native and denatured COMP, which is a large protein that is synthesized by chondrocytes (see also adjuvant-induced arthritis)	▪ Acute and self-limiting polyarthritis in contrast to human RA ▪ Not antigenic but displays an autoimmune pathophysiology	[52,53]
Pristane-induced arthritis (PIA)	Mouse, rat	Injection of the hydrocarbon pristane intraperitoneally into mice	▪ No evidence of autoimmune reactions ▪ Inflammation is restricted to the joints but systemic abnormalities are absent in rats	[47,54,55]
Antigen-induced arthritis (AIA)	Mouse	Inoculation with antigen by intra-articular injection	▪ Intra-articular injection in mice requires advanced technical skills ▪ Does not recapitulate the endogenous breach of tolerance in contrast to human ra ▪ Excludes systemic component of disease	[36,56,57]
Proteoglycan-induced arthritis	Mouse	Intraperitoneal injection of proteoglycan that is emulsified with an adjuvant	▪ Only inducible in susceptible strains of mice ▪ Incidence of ankylosing spondylitis without any exacerbations and remissions in contrast to human RA	[34,35,36]
Glucose-6-phosphate isomerase (G6PI)-induced arthritis	Mouse	Immunization using the ubiquinone containing glycolytic enzyme G6PI with CFA for induction of RA	▪ Only inducible in susceptible strains of mice ▪ Low prevalence of antibodies against G6PI in patients with RA	[34,35,36,58]
***Genetically manipulated spontaneous arthritis models***				
K/BxN model	Mouse	K/B×N mice were generated by crossing mice expressing the MHC class II molecule A^g7^ with the T cell receptor (TCR) transgenic KRN line expressing a TCR specific for a G6PI-peptide	▪ Mutations have only been identified in mice ▪ Low prevalence of antibodies to g6pi in patients with ra ▪ Without systemic manifestations or production of rheumatoid factor in contrast to human RA	[58,59,60]
SKG model	Mouse	Induction of arthritis due to point mutation in ZAP-70	▪ Mutations have only been identified in mice ▪ Disease manifestations in germ-free mice only upon induction	[34,35,36,61]
Human TNF transgene model	Mouse	Transgene for human TNF-α	▪ Mutations have only been identified in mice ▪ No production of rheumatoid factor in contrast to human RA	[34,35,36,62,63]

Therefore, preclinical models are essential to help improve our understanding of pathological mechanisms and to develop and verify new therapeutic approaches with the aim of meeting this unmet medical need. This includes the investigation of human-specific alternatives to identify objective biomarkers to delineate disease subtypes and treatment response, and novel targets to manipulate the function of immune cells involved in the pathogenesis of RA. The purpose of this review was to summarize the most commonly used and often cytokine-based in vitro models of RA, and discuss how they reflect human pathophysiology to further understand the underlying mechanisms of RA.

## 4. Lessons from In Vitro Models of Arthritis: An Alternative without Alternatives

**Figure 2:**
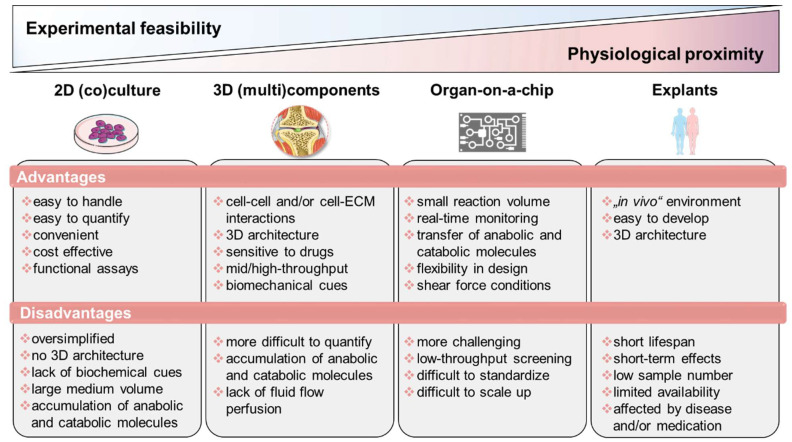
Overview of state-of-the-art in vitro models classified according to experimental feasibility and physiological proximity. Figure contains graphics from Servier Medical Art, licensed under a Creative Common Attribution 3.0 Generic License. http://smart.servier.com/.

During the last decade, promising in vitro techniques have been improved by advances in tissue engineering. Thus, the pathogenesis of RA has been simulated and studied using a variety of in vitro and in vivo models. Cell-based in vitro assays range from tissue explants and relatively simplified (co)-culture systems to complex engineered three-dimensional (multi)component tissue systems using a variety of cell types from cell lines, primary cells, or patient-derived cells, such as mesenchymal stromal cells (MSCs) or pluripotent stem cells (iPSCs), to study, e.g., cell migration, activation, antigen presentation, and cell–cell interaction, as well as cell- and matrix-related changes. Additionally, organoids incubated on microfluidic chips, as well as using in silico models, show promise as an approach to further studying the mechanisms underlying RA pathophysiology and to identify potential new targets. Thus, next-generation preclinical in vitro screening systems will be based on microphysiological in vitro human-joint-on-a-chip systems using primary cells from patients with RA and from different organs, mimicking the systemic nature of the disease and fostering the translational process to humans, while reducing the number of animal experiments. Ultimately, the main goal for all in vitro approaches is to achieve the greatest possible physiological proximity to the disease, while ensuring experimental feasibility, breaking down the barrier to translational medicine and thus to conducting high-quality, reproducible research (Figure 2).

### 4.1. Tissue Explants: Close Physiological Proximity but Low Experimental Feasibility

Ex vivo culture models or tissue explants represent the closest physiological similarity to pathological tissue due to the nature of their origin. If ethically and clinically available, these models can be easily obtained, are easy to develop, and allow the semi-controlled study of the behavior of cells cultured. Although tissue explants reflect the human physiology in terms of 3D structure and environment, they are often affected by individual health status and medication, as well as sample preparation. However, tissue explant approaches are still a powerful tool in, e.g., osteochondral bone research due to the ability to retain native bone cell communication and to study cellular responses and extracellular matrix remodeling processes, including disease-specific matrix degradation in a (patho)physiological bone environment [64]. In addition to their limited availability (especially in terms of healthy human material), the main limitations of tissue explant models are shortened lifespan due to simultaneous disruption of the supplying vessels and, consequently, induced cell death and necrosis-induced cell death at the explant/wound edges [65]. With synovial tissue, explants can be obtained from patients with RA or osteoarthritis (OA) during joint replacement surgery, as well as by needle and arthroscopic biopsy. These types of samples have been comprehensively examined using molecular and immunohistochemical techniques leading to a better understanding of the pathogenic events that occur in the course of the disease [66]. For instance, when studying the association between synovial imaging activity by magnet resonance imaging or color Doppler ultrasound with the expression of synovial inflammatory mediators using tissue explants, Andersen et al. observed a correlation of distinct synovial cytokines with corresponding imaging pathology and disease activity [67].

Samples of synovial [68] and bone explants [69] have been used to study the efficacy and efficiency of therapeutic treatments on the (i) production of pro-inflammatory mediators, (ii) expression of matrix-degrading enzymes, and (iii) adhesion molecules. Of note, IL-1β, TNF-α, and IL-17 have been demonstrated to produce many additive and/or synergistic effects in vitro. Using synovial explants from patients with RA, therapeutic intervention with a combination of biologicals, e.g., anti-TNF-α antibodies and IL-1Ra, resulted in significantly decreased IL-6 and MMP-3 production, indicating the superior efficacy of combinatorial therapy over a single biological treatment [70]. Kirenol, which is a Chinese herbal active component, was demonstrated to inhibit FLS proliferation, migration, invasion, and secretion of pro-inflammatory IL-6 in explants from RA synovium [71].

To examine disease-related expression profiles, explants, like articular cartilage discs, have been obtained from patients with RA after knee arthroplasty. Using this approach, Gotoh et al. demonstrated that the interaction of CD40 with CD154 increased the expression of inflammatory cytokines and MMPs, resulting in an increased cartilage degradation in patients with RA [72]. Based on the aforementioned types of explants, Schultz et al. developed a 3D in vitro model to investigate destructive processes in RA. Although the explant co-culture system did not address all aspects of RA, such as the presence of immune cells, the authors confirmed the capability of their model to study FLS activity on destructive processes of established joint diseases in vitro [73]. More than 10 years later, Pretzel et al. established an in vitro that which closely reflects early processes in cartilage destruction caused by synovial fibroblasts via, e.g., the suppression of anabolic matrix synthesis highlighting the value and close proximity of tissue explant models [9].

### 4.2. Simplified 2D Culture and Co-Culture Approaches for High-Throughput Drug Screening

Closely mimicking physiological and pathophysiological biological complexity in terms of physiological or pathophysiological characteristics requires the use of tissue explants, using 3D architecture or the development of sophisticated complex 3D tissue models. However, achieving experimental feasibility and ensuring adequate nutrient and oxygen supply are more challenging tasks with 3D designs than with 2D cell cultures. Therefore, 2D monolayer cell cultures are a simple and cost-effective alternative, especially for high-throughput screening approaches, which are common in pharmaceutical, industrial, and toxicological research. They are still used to investigate the efficiency and efficacy of therapeutics, to determine their optimal concentration, to analyze disease-related gene expression profiles, and to study cell–cell, cell–microenvironment, or cell–humoral interactions using auto- and paracrine signals, such as in aggregate–cell interactions, in a simplified co-culture system [74,75,76,77]. Two-dimensional monolayer cell cultures are used for rapid in vitro cell expansion, despite the risk of cellular alterations in terms of morphology, genetic alteration, cell diversity, cell cycle progression, and cell differentiation capacity [78]. Accordingly, when 2D modeling cartilage, for instance, the phenotype of chondrocytes becomes unstable, which is indicated by a downregulation of type II collagen (*COL2*) with a simultaneous increase in the expression of type I collagen (*COL1*). To avoid these artificial changes, an optimized cultivation procedure is required using specific plate coatings, such as poly(L-lactic acid) [79]. When investigating the effects of RA-associated cytokines on cartilage, monolayer chondrocyte cultures are considered an optimal tool due to their easy handling in combination with the rapid response of chondrocytes to pro-inflammatory cytokines. In addition, chondrocytes, when stimulated with, e.g., IL-1β, TNF-α, or IFN-γ, show a classical RA-like phenotype as evidenced by decreased expressions of *COL2* and aggrecan (*ACAN*) when *MMP13* expression increases [80] and induced apoptosis in chondrocytes [81], reflecting the human in vivo situation [82,83]. Using the 2D approach, Teltow et al. demonstrated that the majority of IL-1β-treated chondrocytes are produced in collagenase 1 instead of collagenase 3, although the latter has been assumed to foster the destructive processes of RA joints by degrading collagen type II [84]. IL-1β was demonstrated to decrease the expression of *COL2* in 2D monolayer cultures [85].

Expanding the 2D monolayer cultures using co-culture systems, the interaction between cells growing in the same environment can be either indirectly (physical barrier) cultivated by simple medium transfer and using a trans-well chamber or directly cultivated in a mixed culture system providing cell-to-cell contact. Using direct and indirect co-cultivation, Donlin et al. demonstrated that human RA synovial fibroblasts suppress the TNF-α-induced IFN-γ signature in macrophages under both conditions, indicating that no cell contact is required, but rather soluble fibroblast products inhibit the IFN-γ signature of macrophages [86]. To extend the co-culture systems, Pagani et al. developed an advanced tri-culture model to study the interaction between osteoblasts, osteoclasts, and endothelial cells and the cytokine-induced effects on bone homeostasis with respect to RA [87].

### 4.3. 3D tissue Engineering Approaches: Mimicking Structural Features of the Joint

In the field of musculoskeletal disorders, simplified 2D cell culture systems have been stepwise replaced by promising in vitro 3D tissue engineering approaches, including (i) scaffold-free 3D approaches, such as cell-sheet formation [88], self-assembly, or self-organization [89], (ii) natural scaffold-based 3D approaches, such as hyaluronic-acid-based scaffolds [90], and (iii) synthetic scaffold-based 3D approaches, such as poly-(lactide)-based scaffolds [91].

These 3D approaches offer considerable advantages compared to the above-mentioned 2D approaches because they facilitate cell–cell and cell–matrix interactions; cell proliferation, differentiation, and migration and they maintain the cell fate as a result of the physiological 3D structure. To mimic the structural features of the joint, which is a prerequisite for simulating the pathogenesis of RA, the various cell-based components, such as synovial membrane and the chondrogenic and osteogenic parts, must be developed for an in vitro 3D approach.

#### 4.3.1. Synovial Membrane 3D In Vitro Models: From Monolayer to Micromass Culture

The synovial membrane, or synovia, lines the joint cavity and can be divided into the synovial intimal lining (intima) and subintimal lining (subintima). In the healthy state, the intima lining consists of one to four cell layers of type A (macrophages) and type B (FLSs) synoviocytes. The subintimal lining is based on fibrous, areolar, and fatty tissues [92]. As described above, activated FLSs are supposed to be key mediators of joint destruction and drivers of the inflammatory processes during the course of RA. Therefore, FLSs are receiving attention for creating 3D models of the synovial membrane. For this purpose, FLS are resuspended in gels to map a 3D micromass [93]. Karonitsch et al. used such an in vitro 3D micromass model of the synovial membrane to determine the individual effects of pro-inflammatory cytokines, such as IFN-γ and TNF-α, on mesenchymal tissue remodeling [94]. Whereas IFN-γ promotes the invasive potential of FLSs via JAK activation, TNF induces pronounced aggregation of FLSs, indicating that both cytokines affect synovial tissue remodeling in a different manner [94]. Using a similar 3D in vitro approach, Bonelli et al. recently observed that TNF regulates the expression of the transcription factor interferon regulatory factor 1 (*IRF1*), a key regulator of the IFN-mediated inflammatory cascade, which was confirmed by a TNF transgenic arthritis mouse model [95]. Although both studies relied on 3D models solely consisting of FLSs, they indicated that 3D in vitro approaches are sufficient to elucidate mechanistically cellular processes in the FLS-driven inflammation during RA.

Broeren et al. established a sophisticated, promising, and more complex in vitro 3D synovial membrane model by combining either primary RA-FLSs with peripheral CD14+ monocytes or using a complete human RA synovial cell suspension [96]. This model reflects the native 3D architecture of the synovium forming a lining layer at the outer surface consisting of fibroblast-like and macrophage-like synoviocytes. Long-term exposure to TNF-α led to hyperplasia of the lining layer, an altered macrophage phenotype, and an increase in pro-inflammatory cytokines, such as *TNFA*, *IL6*, *IL8*, and *IL1B*, reassembling key features of established RA, thereby confirming previous observations by Kiener et al. [93,96]. The findings of the latter study highlighted the unrestricted possibilities of 3D in vitro approaches to be an excellent alternative for drug testing and mechanistic research.

Although these models closely reflect the inflamed synovial membrane, they all rely on diseased FLSs, which are often limited in availability and are affected by different stages of disease, as well as current medication [97]. To mimic a healthy situation, which is essential to understanding pathogenic alterations of the synovium, an easy to handle and available cell source from different sources that shares properties of FLSs would be ideal for simulating the synovial tissue in vitro. Adult MSCs share most properties with FLSs, including surface markers, differentiation capacity, and the capability to produce hyaluronic acid, and are indistinguishable from each other. Thus, MSCs could be a promising cell source for the development of in vitro 3D models of the synovial membrane or even the other components of the joint [98].

#### 4.3.2. Modeling Articular Cartilage: Scaffold Revisited

To mimic articular cartilage for a 3D in vitro model of arthritis, healthy hyaline cartilage is a relatively acellular and avascular tissue with limited regenerative capacity, nourished by the synovial fluid through diffusion [99]. Articular cartilage is characterized by an organized structure consisting of different layers (superficially tangential, transitional, and radial) that absorbs mechanical loads and forces within the joint and thus protects the underlying subchondral bone. Chondrocytes/-blasts are the only cell population that produce and maintain the highly organized extracellular matrix (ECM), consisting of collagens, mainly type II, type IX, and XI; non-collagen proteins; and proteoglycans, such as aggrecan [99]. During RA, pro-inflammatory stimuli, such as TNF-α or IFN-γ, result in the molecular activation of catabolic and inflammatory processes in human chondrocytes, which decreases their viability and proliferation and increases matrix degradation [81,100].

Due to the sensitivity of chondrocytes to the molecular and mechanical cues of the environment, the consensus is that 3D tissue models, using a matrix that corresponds to the natural tissue properties, are closer to the in vivo situation [101]. Therefore, most 3D approaches involve a scaffold to provide the cells with a predetermined 3D structure. These scaffolds include porous scaffolds made of collagen type II [102], natural gels, such as gelatine microspheres [103], alginate beads [104], hyaluronic acid, and chitosan [105]. Using gelatine microspheres, Peck et al. created a 3D cartilage model very closely mimicking human cartilage, as confirmed by the high expression of type II collagen and proteoglycans [103]. Using a tri-culture approach combining the gelatine microspheres-based 3D cartilage model with a synovial cell line and lipopolysaccharide (LPS)-activated monocytic THP-1 cells, the authors confirmed and validated the pathological alteration in the phenotype of chondrocytes characterized by increased apoptosis, decreased gene expression for matrix components, such as collagen type II and aggrecan, increased gene expression for tissue degrading enzymes (*MMP1*, *MMP3*, *MMP13*, and *ADAMTS4*, *ADAMTS5*) and upregulation of the expression of inflammatory mediator genes (*TNFA*, *IL1B*, and *IL6*), as observed in a disease state of RA [103]. Along this line, stimulation of alginate-based 3D cartilage tissue models with supernatant from RA synovial fibroblasts led to the activation of catabolic and inflammatory processes that could be reversed by anti-rheumatic drugs when used [106]. Ibold et al. developed a 3D articular cartilage model for RA based on the interactive co-culture of high-density scaffold-free porcine cartilage with a RA-derived synovial fibroblast cell line to provide a tool for high-throughput drug screening. For high-throughput purposes, automation of cell seeding was introduced, which improved the quality of the generated pannus cultures as assessed by the enhanced formation of cartilage-specific ECM [107]. However although the stiffness and absorption rate of these natural matrices cannot be adjusted to the specific requirements of each cartilage zone, Karimi et al., modeled the superficial, middle, and calcified zone using varying cell amounts, mechanical loading, and biochemical influences [108].

To establish scaffold-free 3D cartilage constructs, intrinsic processes, such as spontaneous self-assembly, or extrinsic processes, such as mechanical load-induced self-organization, have been described [109,110,111,112] and used as 3D in vitro models for, e.g., preclinical high-throughput screenings [113,114].

Since MSCs, which are progenitors of chondrocytes, can be forced in vitro to enter chondrogenic differentiation, they represent an ideal cell source for the development of in vitro cartilage models: MSCs are available from different tissue sources (even autologous), they are immune privileged, easy-to-handle, and highly expandable. Thus, MSCs have been the focus in numerous studies with and without the incorporation of scaffolds [115,116]. Using this approach, a chondrocyte-like morphology and cartilage-like matrix corresponding to that of native cartilage were reported, particularly with the aim of develop cartilage grafts for therapeutic purposes [115].

#### 4.3.3. The Complexity of Mimicking 3D Subchondral Bone: Mission Impossible?

A key feature of RA is focal bone loss or bone erosion [117]. To address this feature, mimicking bone tissue is mandatory. However, bone tissue is complex in terms of cell composition, matrix organization, vascularization, and mechanical loading. Bone is a dynamic, highly vascularized, and connective tissue that undergoes lifelong remodeling processes in an adaptive response to mechanical stress. It provides a supporting function within the musculoskeletal system and consists of different cell types, such as osteoblasts, osteocytes, and osteoclasts embedded in the ECM, which consists of organic and inorganic phases. Osteoclasts and osteoblasts are key players during bone turnover, whereas osteocytes play a crucial role in bone homeostasis, responsible for mechanosensing and mechanotransduction [118]. Traditionally, bone tissue engineering has been used to produce implants for bone regeneration [119]. In recent years, however, bone tissue engineering has been increasingly applied to create artificial in vitro bone models to improve our understanding of bone-related (patho)physiological mechanisms, such as osteoporosis. Commonly, approaches used to mimic bone in vitro are scaffold-based. Thus, numerous innovative scaffolds (synthetic, natural, biodegradable, and non-biodegradable) have been developed that are capable of mimicking the mechanical stiffness and structural properties of bone; the latter includes mimicking porosity and pore sizes to provide cavities for cell penetration and nutrient supply [120]. These scaffolds are further optimized to have both osteoconductive and osteoinductive properties [121].

Apart from the scaffold-based approaches, scaffold-free organoids or spheroids and 3D printing, hydrogels, or beads have been used [90,122,123,124,125]. However, all of the aforementioned approaches commonly use MSCs capable of differentiating into the osteogenic lineage, osteoblasts, and a combination of either osteoblasts and osteocytes or osteoblasts and osteoclasts. To further support osteogenic properties, bioactive compounds, such as bone morphogenetic protein 2 (BMP-2) or vascular endothelial growth factor (VEGF), have been included [126,127]. To achieve the mechanical impact important for native bone, suitable bioreactors combined with bioceramics further support the in vitro osteogenesis in a defined, standardized, controlled, and reproducible manner [128]. Novel promising approaches aim to realize in vitro bone models with robust vascularization using human umbilical vein endothelial cells [129,130,131]. However, no in vitro 3D bone model is currently available that reflects the complexity of the human bone.

#### 4.3.4. 3D Multicomponent Approaches: Reconstructing the Joint Structure

Multicomponent in vitro 3D co-cultures systems combining 3D in vitro models of articular cartilage and bone (osteochondral unit) with in vitro 3D models of the synovial membrane are necessary to study the cartilage degradation and bone erosive processes during RA that are linked to the invasiveness of the hyperplastic synovium (pannus) [132]. Currently, multicomponent engineering approaches are widely used to simulate key features of osteoarthritis instead of RA or are used to develop suitable artificial matrices that can replace damaged regions and promote tissue regeneration. Thus, many promising in vitro approaches have been recently developed using (i) scaffold-based bone and scaffold-free cartilage [133], (ii) different scaffolds for both bone and cartilage, (iii) a heterogeneous (bi-layered) scaffold, or (iv) a homogenous scaffold for both bone and cartilage (as reviewed in Reference [134]). Notably, bi-layered systems are most often fixed by adhesives, such as fibrin, creating a barrier for cell–cell contact. To avoid this, Lin et al. encapsulated iPSCs-derived MSCs (iMPCs) in a photocrosslinkable gelatin scaffold. Using a dual-flow bioreactor, encapsulated iMPCs were chondrogenic (top) and osteogenic (bottom) differentiated to directly form a stable bridging zone between the both tissue models [135]. So far, no appropriate multicomponent in vitro model exists that is able to mimic the physiologically relevant environment of a healthy or an inflamed joint, including all signaling molecules, cells, and tissue types. Consequently, we developed a valid in vitro 3D model to simulate the immune-mediated pathogenesis of arthritis. The in vitro model relies on the three main components of the joint: (i) the osteogenic and (ii) chondrogenic parts, and (iii) the synovial membrane with the synovial fluid. All components are based on differentiated MSCs from a single donor and thus include most relevant cell types involved, enabling crucial cell–cell interactions [136]. We simulated the inflamed joint using the application of RA-related cytokines, as well as immune cells [132]. Finally, we confirmed the suitability of the multicomponent in vitro 3D model, which may serve as a preclinical tool for the evaluation of both new targets and potential drugs in a more translational setup [137].

## 5. Microfluidic Approaches: Prospectively Systemic

**Figure 3:**
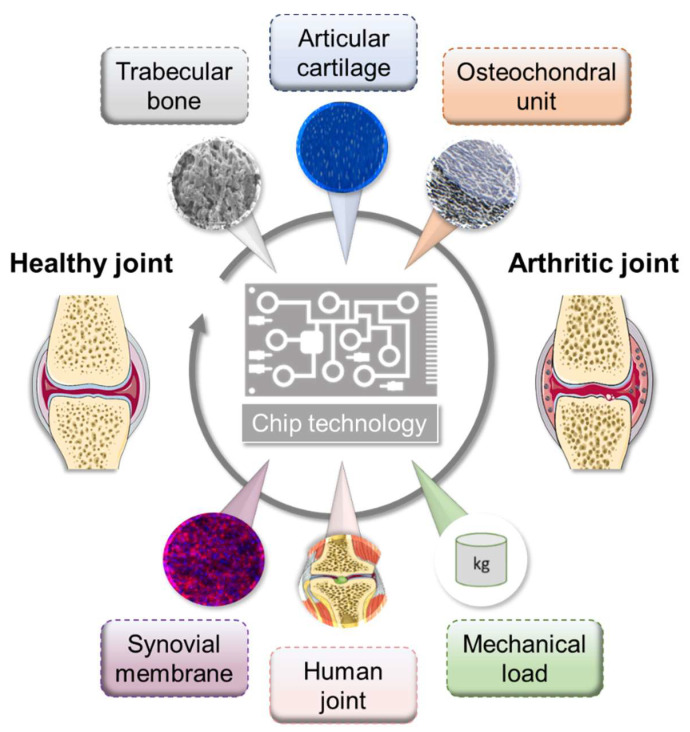
Overview of microfluidic approaches mimicking selected physiological interactions of the human joint tissues. Figure was modified from Servier Medical Art, licensed under a Creative Common Attribution 3.0 Generic License. http://smart.servier.com/.

In recent years, perfused cultivation systems have become increasingly important due to the advantages they provide for the cultivation of functional tissues. They ensure the permanent supply of nutrients and the defined real-time monitoring of environmental conditions, such as pH, temperature, and oxygen concentrations. Multi-chamber bioreactors provide the opportunity to cultivate two or more cell/tissue types in a defined manner [138,139]. Generally, microfluidic approaches provide inherent flexibility in combinatory design, which enables relevant concentration gradients, cellular spatial configuration, and co-culture and shear force conditions [140]. To date, only a few different microfluidic culture approaches have been reported that at least partially reflect the physiology of the joint structure, mimicking either subchondral bone, articular cartilage, or both together, namely the osteochondral part, as well as the synovial membrane, including spatial topology and mechanical loading [141,142]. However, these do not yet cover all the possibilities offered by these microfluidic systems (Figure 3).

In detail, using equine chondrocytes in a microfluidic culture, 3D cartilage constructs were formed by establishing a physiologic nutrient diffusion gradient across a simulated matrix. Additionally, the geometric design constraints of the microchambers drive native cartilage-like cellular behavior [141]. Calvo et al. developed a synovium-on-a-chip system by culturing patient-derived primary FLSs in a Matrigel^TM^-based 3D micro-mass mimicking TNF-α-driven structural changes and synovial remodeling [142]. As a result, the activation of FLSs by TNF-α leads to induction of the expression of pro-inflammatory cytokines, such as *IL6* and *IL8*, as well as matrix-degrading metalloproteinases and pannus formation, which is a typical feature of RA. Since the rea-out parameters in a perfused system are often limited to endpoint assessments, the chip system reported by Calvo et al. (2017) facilitates the online monitoring of cellular parameters by incorporating a simplified light scattering method that enables the non-invasive detection of cell motility, proliferation, invasion, and even matrix condensation processes within the 3D tissue [142].

Conclusively, the ultimate goals of microfluidic approaches are to (i) provide reliable information on the health and disease status of the integrated complex biological system, (ii) reproducibly promote the formation of the microphysiological tissue structure, and (iii) non-invasively and automatically monitor stimuli-driven tissue responses [143].

**Figure 4:**
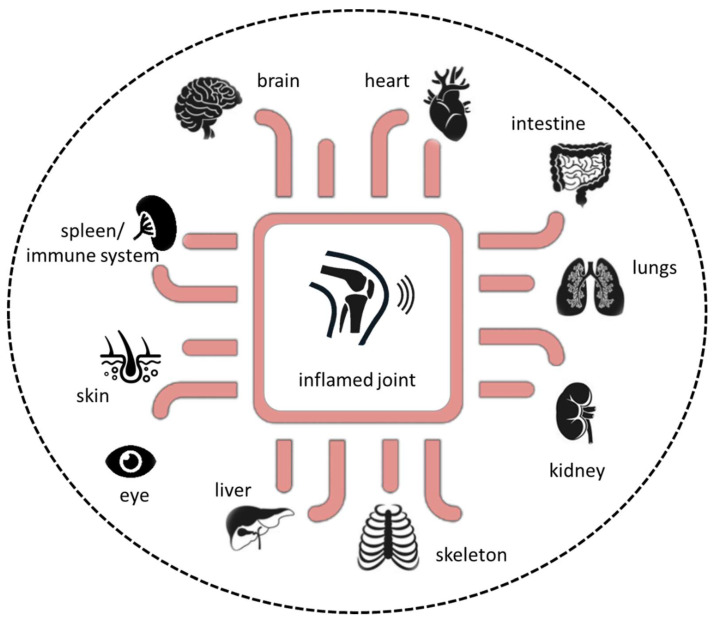
Next-generation preclinical in vitro approach based on microphysiological in vitro human-joint-on-a-chip systems in combination with pathophysiological-relevant human organs.

Additionally, a variety of organoids representing various tissues, such as liver, kidney, or heart, have been established and implemented in microfluidic systems as a single-tissue approach, namely organ-on-a-chip, or as multi-tissue approaches, such as multi-organ-on-a-chip or, if possible, human-on-a-chip (Figure 4). However, the human-joint-on-a-chip approach could be a promising in vitro tool to improve our understanding of the complex pathophysiological mechanisms in RA and to develop and verify new therapeutic strategies to further expand our repertoire in the battle against this potentially devastating disease. Future perspectives include human-joint-on-a-chip tailored to a single patient for use in a personalized medicine scenario to maintain human health.

Despite their advances and opportunities for translational studies and drug testing, microfluidic systems have still some limitations. So far, microfluidic systems are more challenging to operate and control than static systems, some organ functions, such as cognition on the brain and mechanical function in bone, cannot be readily modeled, and they are difficult to adapt to high-throughput screening and are difficult to standardize and scale up.

## 6. Conclusions and Outlook

Here, we comprehensively summarized key events in RA pathogenesis, which is the most common immune-mediated chronic inflammatory joint disease. Today’s treatment goal of RA is to achieve remission or at least low disease activity. However, a strong unmet medical need remains, as by far not all patients reach sustained clinical remission and even about 25% still suffer from moderate or even high disease activity characterized by systemic inflammation, persistent synovitis, expansion of synovial cells (pannus formation) and progressive cartilage and bone destruction in late stages. In the last years, we have witnessed the failure of potential new therapies in clinical trials although their development was based on promising preclinical animal data, which can be attributed to the nature of these models. Animal models and simplified 2D cell cultures of arthritis have been useful to identify certain pathomechanisms underlying RA. However, they do not fully reflect human pathogenesis due to oversimplification of the pathophysiological processes or misleading in case of animal models which owe interspecies differences with regard to, e.g., chondrocyte biology, articular cartilage, and cartilage thickness [144,145,146].

Thus, we herewith suggest that shifting our traditional research approaches in biomedicine towards an improved human personalized patient-driven translation by using sophisticated in vitro models may enhance ‘precision’ in medicine. Finally, personalized in vitro models will provide guidance to replace today’s inefficient standard treatment regimens (one fits it all) taking into account patient heterogeneity in terms of disease subtypes, endogenous circadian rhythms, autoantibodies, cytokine and infiltrating immune cell patterns, and the extent of pannus formation, ultimately preventing ‘refractory’ arthritis.

Along with the joints, RA can affect many of the body’s organs [2,3]. Therefore, combining different 3D tissue models with state-of-the-art microfluidic devices must be the next generation in vitro approach to study the complex crosstalk between tissues/organs and the immune system, including the spreading of (auto)immune reactions across different organs, ultimately mimicking the systemic nature of rheumatic diseases.

Prospectively, the human-based approach will not only provide opportunities (i) to identify objective patient-related biomarkers to elucidate disease subtypes and treatment response but also (ii) enable strategies for the management of patients who are ‘refractory’ or resistant to available treatments. Thus, human-based cellular and tissue models will close the gaps in RA research and, finally, health care, increase clinical translatability, and contribute to the reduction and/or replacement of animal experiments used in basic and translational RA research.
